# Characteristics of patients attending the child and adolescent psychiatric outpatient clinic in Erbil city

**DOI:** 10.1371/journal.pone.0209418

**Published:** 2019-02-28

**Authors:** Sahar Adnan Abdulqader, Banaz Adnan Saeed

**Affiliations:** 1 Erbil Psychiatric Hospital, Erbil, Iraq; 2 Department of Psychiatry, College of Medicine, Hawler Medical University, Erbil, Iraq; Sapienza University fo Rome, ITALY

## Abstract

**Background:**

The Erbil psychiatric hospital is the major governmental psychiatric facility in the governorate of Erbil, the capital of the Kurdistan region in Iraq, providing services for the diagnosis, treatment and follow-up of patients from the city and its surrounding areas. The child and adolescent outpatient clinic in the hospital is for patients younger than 18 years. The objectives of this study were to explore the sociodemographic, pregnancy and birth characteristics, as well as the clinical profiles, of patients who presented to the child and adolescent psychiatric outpatient clinic to statistically estimate the frequency of various psychiatric disorders among the attendees and to test hypotheses of the association of these psychiatric disorders with sociodemographic and birth and pregnancy characteristics as reported by many previous studies but with inconsistent results.

**Methods:**

A descriptive consecutive cross-sectional study was conducted from October 2017 to February 2018 in the Child and Adolescent Psychiatric Outpatient Clinic in Erbil province of Iraq’s Kurdistan region. The diagnostic criteria of the DSM-5 (Diagnostic and Statistical Manual of Mental Disorders, 5^th^ edition) were implemented. Chi-square tests were used to test the hypothesized associations.

**Results:**

Of a total of 207 patients, 142 were males and 65 were females, and most lived in low socioeconomic status. Most of the patients’ fathers were school educated, and most mothers were illiterate. Consanguinity was found in 41% of the parents, 26.6% of the patients were born by caesarean section(C/S), 62.8% were nurtured exclusively by breastfeeding in their first six months after birth, 42.5% of them visited faith healers before consulting a psychiatrist, 43% had neurodevelopmental disorders, 26.6% had intellectual disabilities with behavioral and emotional comorbidities and 30.4% had other mental/behavioral disorders.

**Conclusion:**

This study revealed that the child and adolescent psychiatric disorders in Erbil city are versatile and that many factors are significantly associated with them. Cultural concepts are still influential in the region in a way that can interfere with children’s well-being, a situation that calls for more concern and attention.

## Introduction

According to the World Health Organization (WHO), 10–20% of children and adolescents experience mental disorders worldwide[1], and the roughly estimated prevalence in Iraq and other countries in the region is 10–36%, which is significantly higher than the estimates for developed countries[2]. These disorders are the leading cause of disability in young people in all regions. If untreated, they severely influence children’s development, their educational attainments and their potential to live fulfilling and productive lives. Children with mental disorders face significant challenges with stigma, isolation, and discrimination, as well as a lack of access to health care and education facilities, in violation of their fundamental human rights[1]. Moreover, the regional conflicts and war exposure has had an additional impact on children’s lives. These conflicts have split the children’s communities and taken the lives of hundreds of thousands of their friends and family members[3]. Erbil is the capital of the Kurdistan region in Iraq and is one of the most populous cities in northern Iraq. To the best of our knowledge, there are no data about the characteristics of children and adolescents attending outpatient clinics in the Kurdistan region of Iraq in the last few years. This study aimed to shed light upon patients attending the child and adolescent psychiatric outpatient clinic at Erbil Psychiatric Hospital through documenting their psychiatric disorders and studying the association of these disorders with some of the commonly purported parental, perinatal and sociodemographic factors.

## Patients and methods

The design was a consecutive cross-sectional study; participants were children and adolescents attending the Child and Adolescent Psychiatric Outpatient Clinic in Erbil Psychiatric Hospital.

Erbil psychiatric hospital is the primary governmental psychiatric facility in the governorate of Erbil providing services of diagnosis, treatment and routine follow-up to patients who come from the city, its surroundings and displaced immigrants from all over the region who settled there searching for peace due to the Islamic State in Iraq and Syria (ISIS) crises; it is the only hospital that has a child and adolescent psychiatric unit in the Kurdistan Region of Iraq. Assessment of consecutive cases was performed during the period from October 1, 2017, to January 31, 2018, and included all the patients who visited the clinic during the time of the study. The exclusion of patients only occurred when the patient or his/her parent refused to take part in the study.

### Ethical agreement

Prior to data collection, official written ethical approval was obtained from the Research Ethics Committee of Hawler Medical University. Initial informed verbal consent was obtained from the parent of the patient, after an explanation of the aims and the objectives of the study, and then the patients were asked for their consent to participate in the research; in cases with very young age children, severe intellectual disability, severe ASD or a communication disorder where the patient could not understand what consent meant, we had to depend on the parent consent to make sure that the child or the adolescent was comfortable going through the steps of the research and assessment.

The local Research Ethics Committee approved this consent procedure after considering the following points:

Written consent, if it was to be considered, would not apply to all participants and parents. Verbal consent was assumed to be understood by illiterate and partially illiterate participants and parents. Moreover, written consent would have involved the officialdom, which raised skepticism and cultural issues by some of the participants. The information sheet and written consent form were available and were used as a guide to obtain the verbal informed consent by explaining several points to the parent and the patients in their native language (Kurdish, Arabic and others).

### Study procedure

Before the initiation of this study, formal administrative approval was obtained. The researcher introduced herself to the participants and their families and explained the study aims. The researcher conducted the interviews in a private room. Subjects were given assurance of confidentiality. The researcher interviewed the child and the accompanying relative separately. Case files for each patient were examined for any additional information, such as notes on previous visits and follow-ups.

### Methods of assessment

The following data on sociodemographic and clinical factors were collected during the clinical interview.

**Sociodemographic information**: This information included sex, date of birth, the season of birth, religion, ethnicity, residence, educational status and patient’s birth order among the siblings.

**Family characteristics**: These characteristics included family size, family type, residential area and socioeconomic status. Information regarding the age, education, and occupation of both parents, as well as consanguinity between them, was also obtained.

**Pregnancy and birth information**: Information about antenatal care and follow-up, mode of delivery, birth weight, gestational age at birth, history of admission to the neonatal intensive care unit (NICU) and feeding method in the first six months after birth was collected.

**Medical and psychiatric history**: Information about the chief complaint, duration of illness, source of referral, visits to faith healers before psychiatric consultation, history of previous visits to the psychiatric outpatient clinic, family history of mental illness, past psychiatric history, past medical history and the child’s general developmental level was collected. The child’s general developmental levels were assessed by the General Developmental Scale (GDS) in those patients who were suspected to have a borderline or below age development by their families, school staff or the medical staff, as in cases of intellectual disabilities or neurodevelopmental disorders.

The GDS is a summary scale that provides an overall index of development consisting of the most age-discriminating items from the other scales. It consisted of 10 items from a scale assessing social function, 10 items for self-help, 10 for gross and 10 for fine motor skills, 10 items for expressive language and 10 for language comprehension, and 5 items from the letter and 5 from the number scales. For interpreting the scores, up to 30% below age expectations, i.e., means within 2 SD (standard deviation) below the expected score, was considered borderline, and >30% below age expectations, i.e., means >2 SD below expected, indicated delayed development[4].

**Diagnosis and treatment management**: For psychiatric diagnosis, we relied on the Diagnostic and Statistical Manual of Mental Disorders, 5^th^ edition[5]. Detailed information about the management and treatment plans (pharmacological, psychological or both) was registered.

### Association testing

We tested the hypothesis of an association between children’s mental disorders and sociodemographic, birth and pregnancy characteristics. For this purpose, the disorders were categorized into three main groups as follows:

- The neurodevelopmental disorders group included patients with intellectual disabilities (IDs), autism spectrum disorder (ASD), communication disorders, specific learning disorders and attention-deficit hyperactivity disorder (ADHD).
- The comorbid disorder group included patients who were already diagnosed with IDs and presented with behavioral, emotional, anxiety or other problems.
- The other mental/behavioral disorders group included all other disorders, such as conduct disorders, generalized anxiety disorder (GAD), major depressive disorder (MDD), trauma- and stressor-related disorders, conversion disorders, relational problems, psychotic disorders, phobias, obsessions and others.

The rationale behind the subgroups (neurodevelopmental, comorbid disorders, other behavioral/emotional disorders) was the need to summarize our diagnoses in groups to be able to test associations with the factors, and the fact was that the data showed a dominance of the neurodevelopmental disorders, according to criteria outlined by DSM-5[5], which outnumbered other disorders combined. Regarding the comorbid disorders; we found that the intellectual disabilities cases came mostly for comorbidity reasons, such as mood or anxiety disorders, and they did not fit into other categories exclusively. These three groups were tested for associations with important characteristics such as sex, age group, season of birth, educational level of the patient, residence, birth order in family, socioeconomic status, family size, paternal and maternal age, education and consanguinity between them, family history of mental disorders, history of abortions, antenatal care, mode and place of delivery, gestational age at birth, birth weight, active or passive smoking during pregnancy, history of infertility, diabetes in pregnancy and any other medical conditions that were reported by the mothers.

### Statistical procedure

Data coding and analysis were performed using the Statistical Package of the Social Sciences (SPSS version 23), and frequencies and percentages were used to express categorical variables. Intervals, means and standard deviations were used to express continuous numerical data. The chi-square test was used to test the statistical hypothesis of an association and its significance. Fisher’s exact test was used in cases where more than 20% of the expected frequencies were less than 5.

Associations were considered statistically significant when p ≤0.05.

Table 1 shows the individual sociodemographic characteristics of the patients; more than 68% were males. Their ages ranged from 2–18 years. The age group (6–9) years represented 39% of the cases. As a result, most of the patients (38.2%) were primary school students. Winter was the most common season of birth. Most of the patients were Muslims and Kurds; only 17% were Arabs, and they were mostly immigrants from nearby areas. An urban residence was the most common, and only 2.4% were from refugee camps. Most of the patients were first-born children.

**Table 1:** Sociodemographic characteristics (individual characteristics).

(n = 207)		No.	%
**Sex**	Male	142	(68.8)
	Female	65	(31.4)
**Age group (years)**	2–5	43	(20.8)
	6–9	81	(39.1)
	10–13	47	(22.7)
	14–18	36	(17.4)
**Season of birth**	Winter	71	(34.3)
	Spring	42	(20.3)
	Summer	55	(26.6)
	Autumn	39	(18.8)
**Religion**	Muslim	205	(99.0)
	Christian	2	(1.0)
**Ethnicity**	Kurd	188	(90.8)
	Arab	17	(8.2)
	Others	2	(1.0)
**Residence**	Urban	152	(73.4)
	Suburban	46	(22.2)
	Rural	4	(1.9)
	Refugee camp	5	(2.4)
**Educational level**	Preschool age	55	(26.6)
	Primary stage	79	(38.2)
	Intermediate stage	15	(7.2)
	Secondary stage	7	(3.4)
	Quit school	14	(6.8)
	Never registered	37	(17.9)
**Birth order**	1^st^	76	(36.7)
	2^nd^	44	(21.3)
	3^rd^	28	(13.5)
	4^th^-11^th^	59	(28.5)

Table 2 shows the family sociodemographic characteristics; the mean family size was 6.5± 2.8 with a minimum of 3 and a maximum of 28 members, and the mode was 5 members. Most of them were nuclear families. The residential area ranged from (50–500) m^2^ with a mean of 150±67 m^2^ and a mode of 100 m^2^. Low socioeconomic status was predominant. Paternal age ranged from (17–58) years with a mean of 31.5±8.3 and a mode of 25 years. Maternal age ranged from (15–46) with a mean of 27.6±7 and a mode of 24 years. Fathers were mostly school educated with illiteracy in only 21.3% of them. In mothers, illiteracy dominated, and only 35.7% were school educated. The number of fathers who supported their families by daily wages and those who had a monthly salary were almost equal, and only 4.8% were not supporting their families. Most mothers were housewives. Consanguinity was found in 41% of the parents.

**Table 2:** Sociodemographic characteristics (family characteristics).

(n = 207)		No.	%
**Family size: mean± SD**	6.5 ± 2.8		
**Family type**	Nuclear	179	(86.5)
	Joint	28	(13.5)
**Residential area: mean± SD (m**^**2**^**)**	150± 67		
**Socioeconomic status**	Low	117	(56.5)
	Medium	89	(43.0)
	High	1	(0.5)
**Paternal age: mean ±SD**	31.5 ± 8.3		
**Maternal age: mean ±SD**	27.6 ± 7		
**Father’s education**	Illiterate	44	(21.3)
	Reads and writes	31	(15)
	School education	105	(50.7)
	Higher education	27	(13)
**Mother’s education**	Illiterate	87	(42.0)
	Reads and writes	28	(13.5)
	School education	74	(35.7)
	Higher education	18	(8.7)
**Father’s occupation**	Unemployed	8	(3.9)
	Wage-earner	94	(45.4)
	With a salary	95	(45.9)
	Out of picture (divorced or deceased)	10	(4.8)
**Mother’s occupation**	Housewife	187	(90.3)
	Wage-earner	5	(2.4)
	Employee	15	(7.2)
**Consanguinity between parents**	No	122	(58.9)
	Yes	85	(41.1)

Table 3 shows the pregnancy and birth characteristics: most of the mothers had proper antenatal care, and 26% of them had no proper regular checkups during pregnancy. A total of 73.4% of the patients were delivered by normal vaginal delivery (NVD), and more than 20% of those were delivered outside a healthcare facility. Most children were born as term babies with average birth weight, and 14.5% were admitted to the NICU at birth for different reasons. Exclusive breast feeding in the first 6 months was found in 62.8% of the patients.

**Table 3:** Pregnancy and birth characteristics.

(n = 207)			No.	%
**Proper antenatal care**	No		54	(26.0)
	Yes		153	(74.0)
**Mode of delivery**	NVD	At hospital	120	(73.4)
		Outside healthcare facility (by midwife)	32	
	C/S: causes	Dystocia (failure to progress)	20	(26.6)
		Previous Cesarean Section	14	
		Maternal conditions	9	
		Abnormal fetal presentation	6	
		Placenta Previa	2	
		On request	2	
		Fetal congenital anomaly	1	
		Fetal distress	1	
**Birth weight**	Below average		25	(12.1)
	Average		179	(86.5)
	Above average		3	(1.4)
**Gestational age at birth**	Preterm		14	(6.8)
	Term (37–42)		187	(90.3)
	Post term		6	(2.9)
**NICU Stay**	No		177	(85.5)
	Yes		30	(14.5)
**Feeding method (first 6 months after birth)**	Breastfeeding		130	(62.8)
	Bottle feeding		58	(28.0)
	Mixed feeding		19	(9.2)

Table 4 shows the medical and psychiatric history. The most common chief complaint that led patients to visit the clinic was speech problems, and fewer complaints were about suicidal attempts and substance misuse. The duration of the illness ranged from 2 weeks-60 months. Most of the patients had an illness for more than a year. Forty percent were referred from nonpsychiatric clinics, and only 5.8% were referred from psychiatric clinics; 42.5% of the families visited a faith healer prior to their consultation, and most of those did so more than once. The mean number of psychiatric visits was 4±6 visits, which reached as many as 36 visits in chronic cases, and 58.5% had no family history of mental disorders. Concerning the general development of the patients, 29.5% were within their age expectation, 18.8% scored within 2 SD below their chronological age expectation, placing them in the borderline development group according to the scale, and 51.7% scored more than 2 SD below age, making them delayed compared to their peers.

**Table 4:** Medical and psychiatric history of the patients.

(n = 207)			No.	%
**Chief complaint**	Speech problems		43	(20.8)
	Disturbed behavior		41	(19.8)
	Hyperactive, chaotic behavior		39	(18.8)
	Age inappropriate behavior		17	(8.2)
	Poor school performance		16	(7.7)
	Physical complaints (SOB, chest pain, etc.)		9	(4.3)
	Inappropriate social interaction		9	(4.3)
	Self-injurious behavior		9	(4.3)
	Abnormal movement or posturing		8	(4.0)
	Self-wetting or soiling		4	(2.0)
	Suicidal thoughts or attempts		3	(1.4)
	Obsessional thinking		3	(1.4)
	Fear feelings		3	(1.4)
	Substance misuse and others		3	(1.5)
**Duration of present illness**	<1 month		5	(2.5)
	1–6 months		50	(24.0)
	6–12 months		37	(17.8)
	>12 months		115	(55.6)
**Pathway of first consultation or referral**	Direct		73	(35.3)
	Psychiatric clinic		12	(5.8)
	Other clinics		82	(39.6)
	School or nursery administration		24	(11.6)
	Others (dispensary, ER, camps)		16	(7.7)
**Visits to faith healers before consultation**	No		119	(57.5)
	Yes		88	(42.5)
**History of psychiatric visits: mean ± SD**	4.1 ± 6.1			
**Family history of mental disorders**	No		121	(58.5)
	In 1^st^ degree relative	In father	5	(15.0)
		In mother	7	
		In sibling	19	
	In 2^nd^ degree relative		18	(8.7)
	In 3^rd^ degree relative		22	(10.6)
	In > than one relative		15	(7.2)
**Past psychiatric history**	No		162	(78.2)
	Yes		45	(21.8)
**Past medical history**	No		157	(75.8)
	Yes		50	(24.2)
**GDS scores**	Within age expectations		61	(29.5)
	Borderline (within 2 SD below age expectations)		39	(18.8)
	Delayed (>2 SD below age expectations)		107	(51.7)

Table 5 illustrates diagnosis and treatment management. The diagnosis was dominated by the IDs in 44% of the patients, 17.4% of the IDs cases had comorbidities such as behavioral and emotional problems, anxiety and others. ASD was diagnosed in 17.4% of the patients. In the management plan, most of the patients (43.5%) received solely psychotherapy, and only 13% were managed exclusively with pharmacological treatment; 43.5% of them needed combined treatment (pharmacological and psychological). The table describes the pharmacological treatment types and percentages in detail.

**Table 5:** Diagnosis and treatment management.

**Psychiatric disorders**	IDs with comorbid psychiatric disorders		55	(26.6)
	IDs		36	(17.4)
	ASD		36	(17.4)
	Conduct disorder		12	(5.8)
	Communication disorders		10	(4.8)
	GAD		9	(4.3)
	MDD		9	(4.3)
	Trauma and stressor related disorders		5	(2.4)
	Conversion disorder		5	(2.4)
	Enuresis		4	(1.9)
	ADHD		4	(1.9)
	Parent-child relational problem		4	(1.9)
	Specific learning disorder		3	(1.4)
	Bipolar I disorder		2	(1.0)
	Grief reaction		2	(1.0)
	Schizophreniform disorder		2	(1.0)
	Panic disorder		2	(1.0)
	Obsessive-compulsive and related disorders		2	(1.0)
	Opioid related disorders		2	(1.0)
	Phobia		1	(0.5)
	Suicidal behavior disorder		1	(0.5)
	Child physical and psychological abuse		1	(0.5)
**Management**	Psychological		90	(43.5)
	Pharmacological		27	(13.0)
	Both		90	(43.5)
**Pharmacological treatment (n = 117)**	Antipsychotics	Atypical	46	(40.2)
		Traditional	1	
	Antidepressants	SSRI	17	(25.6)
		TCA	13	
	Stimulants		0	(0.0)
	Atomoxetine		8	(6.8)
	Vitamins and supplements		6	(5.0)
	More than 1 medication		20	(17.0)
	More than 2 medications		6	(5.0)

Table 6 shows the results of the chi-square association test between sociodemographic variables (individual characteristics) and diagnoses; age group and educational status were significantly associated with the 3 subgroups of diagnoses (neurodevelopmental disorders, IDs with comorbidities and other mental/behavioral disorders). Residence was significantly associated with the latter two, while season of birth was only significantly associated with neurodevelopmental disorders. Neither sex nor the order of birth were significantly associated with the disorders.

**Table 6:** Chi-square association tests between sociodemographic variables (individual characteristics) and diagnoses.

	Neurodevelopmental disorders ^a^		IDs with comorbidities ^b^		Other mental/behavioral disorders ^c^	
		n%		n%		n%
**Sex**	P = 0.3		P = 0.6		P = 0.1	
Male		64 (45)		39(27.5)		39(27.5)
Female		25(38.5)		16(24.5)		24(37.0)
**Age groups**	**P< 0.001**		**P = 0.01**		**P< 0.001**	
2–5		31(72)		5(11.6)		7(16.3)
6–9		45(55.5)		19(23.5)		17(21.0)
10–13		11(23.4)		18(38.3)		18(38.3)
14–18		2(5.5)		13(36.1)		21(58.3)
**Season of birth**	**P = 0.02**		P = 0.3		P = 0.2	
Winter		21(29.6)		24(33.8)		26(36.6)
Spring		21(50.0)		11(26.2)		10(23.8)
Summer		25(45.5)		11(20.0)		19(34.5)
Autumn		22(56.4)		9(23.0)		8(20.5)
**Educational status**	*P<0.001		*P<0.001		*P<0.001	
Preschool age		39(71.0)		8(14.5)		8(14.5)
Primary school		34(43.0)		17(21.5)		28(35.4)
Intermediate school		1(6.6)		2(13.3)		12(80.0)
Secondary school		0(0.0)		0(0.0)		7(100.0)
Quit school		1(7.2)		7(50.0)		6(42.8)
Never registered		14(37.8)		21(56.7)		2(5.4)
**Residence**	*P = 0.2		*P = 0.009		*P = 0.006	
Urban		69(45.4)		32(21.0)		51(33.5)
Suburban		18(39.1)		21(45.6)		7(15.2)
Rural		2(50.0)		1(25.0)		1(25.0)
Refugee camp		0(0.0)		1(20.0)		4(80.0)
**Birth order**	P = 0.1		P = 0.08		P = 0.8	
1^st^		37(48.6)		15(19.7)		24(31.6)
2^nd^		22(50.0)		10(22.7)		12(27.3)
3^rd^ or more		30(34.5)		30(34.5)		27(31.0)

*Fisher’s Exact Test **p value ≤ 0.05**: statistically significant association

^a^ Neurodevelopmental disorders group with n = 89 (43%)

^b^ IDs with comorbidities group with n = 55 (26.6%)

^c^ Other mental/behavioral disorders group n = 63 (30.4%)

Table 7 shows the results of the chi-square association test between sociodemographic variables (family characteristics) and diagnoses. Socioeconomic status was significantly associated with neurodevelopmental disorders and IDs with comorbidities. Parents’ education and consanguinity between them were significantly associated with IDs with comorbidities. Other factors, such as family size, paternal and maternal age, family history of mental disorders and history of abortions, had no significant association with the diagnoses.

**Table 7:** Chi-square association test of sociodemographic variables (family characteristics) and diagnoses.

	Neurodevelopmental disorders ^a^		IDs with comorbidities ^b^		Other mental/ behavioral disorders ^c^	
		n%		n%		n%
**Socioeconomic status**	*P = 0.003		*P = 0.02		*P = 0.2	
Low		40(34.2)		39(33.3)		38(32.5)
Medium		49(55.0)		16(18.0)		24(27.0)
High		0(0.0)		0(0.0)		1(100.0)
**Family size**	P = 0.08		P = 0.3		P = 0.2	
≤4		23(54.7)		9(21.4)		10(23.8)
≥5		66(40.0)		46(28.0)		53(32.0)
**Paternal age**	P = 0.2		P = 0.06		P = 0.4	
≤ 20		2(22.2)		3(33.3)		4(44.4)
21–30		42(40.4)		35(33.6)		27(26.0)
31–40		27(43.5)		13(21.0)		22(35.5)
≥ 41		18(56.3)		4(12.5)		10(31.2)
**Father’s education**	*P = 0.1		**P = 0.05**		P = 0.2	
Illiterate		17(38.6)		15(34.0)		12(27.3)
Reads and writes		10(32.2)		13(42.0)		8(25.8)
School education		46(43.8)		21(20.0)		38(36.2)
Higher education		16(59.2)		6(22.2)		5(18.5)
**Maternal age**	P = 0.6		P = 0.8		P = 0.4	
≤ 20		13(39.3)		8(24.3)		12(36.3)
21–30		48(44.4)		27(25.0)		33(30.5)
31–40		22(39.3)		17(30.3)		17(30.3)
≥ 41		6(60.0)		3(30.0)		1(10.0)
**Mother’s education**	*P = 0.2		**P = 0.02**		P = 0.7	
Illiterate		32(36.8)		29(33.3)		26(29.9)
Reads and writes		11(39.3)		9(32.1)		8(28.6)
School education		35(47.9)		17(23.3)		21(28.8)
Higher education		11(57.9)		0(0.0)		8(42.1)
**Consanguinity between parents**	P = 0.3		**P = 0.007**		P = 0.1	
No		56(45.9)		24(19.7)		42(34.4)
Yes		33(38.8)		31(36.5)		21(24.7)
**Family history of mental disorders**	P = 0.1		P = 0.9		P = 0.1	
No		57(47.1)		32(26.4)		32(26.4)
Yes		32(37.6)		23(26.0)		3(36.4)
**History of abortions**	P = 0.3		P = 0.4		P = 0.09	
No		56(45.5)		35(28.4)		32(26.0)
Yes		33(39.2)		20(23.8)		31(37.0)

*Fisher’s Exact Test **p value ≤ 0.05**: statistically significant association

^a^ Neurodevelopmental disorders group with n = 89 (43%)

^b^ IDs with comorbidities group with n = 55 (26.6%)

^c^ Other mental/behavioral disorders group n = 63 (30.4%)

Table 8 illustrates the results of the chi-square association test between pregnancy and birth characteristics and diagnoses; the mode of delivery had a significant association with neurodevelopmental disorders and IDs with comorbidities. Gestational age at delivery was significantly associated with neurodevelopmental disorders, while diabetes in pregnancy had a significant association with IDs and comorbidities. Passive and/or active smoking in pregnancy was significantly associated with other mental/behavioral disorders. Other factors, such as antenatal care, place of NVD, birth weight and others, showed no significant association with the diagnoses.

**Table 8:** Chi-square association test of pregnancy and birth characteristics and diagnoses.

	Neurodevelopmental disorders ^a^		IDs with comorbidities ^b^		Other mental/ behavioral disorders ^c^	
		n%		n%		n%
**Antenatal care**	P = 0.3		P = 0.3		P = 0.8	
No		20(37.0)		17(31.5)		17(31.5)
Yes		69(45.0)		38(24.8)		46(30.0)
**Mode of delivery**	**P = 0.01**		**P = 0.04**		P = 0.5	
NVD		58(38.2)		46(30.2)		48(31.6)
C/S		31(56.3)		9(16.3)		15(27.3)
**Place of NVD**	P = 0.08		P = 0.1		P = 0.7	
Hospital		50(41.6)		33(27.5)		37(30.8)
Home		8(25.0)		13(40.6)		11(34.3)
**Gestational age at delivery**	*P = 0.02		*P = 0.6		*P = 0.2	
Preterm Baby		9(64.3)		2(14.3)		3(21.4)
Term Baby		75(40.1)		52(27.8)		60(32.1)
Post term Baby		5(83.3)		1(16.7)		0(0.0)
**Birth weight**	*P = 0.9		*P = 0.09		*P = 0.5	
Below Average		10(40.0)		9(36.0)		6(24.0)
Average		78(43.6)		44(24.6)		57(31.8)
Above Average		1(33.3)		2(66.6)		0(0.0)
**Smoking(passive and/or active)**	P = 0.08		P = 0.7		**P = 0.03**	
No		58(47.9)		33(27.3)		30(24.8)
Yes		31(36.0)		22(25.6)		33(38.4)
**Pregnancy after>1year of infertility**	P = 0.1		P = 0.9		P = 0.1	
No		76(41.3)		49(26.6)		59(32.1)
Yes		13(56.5)		6(26.1)		4(17.4)
**Diabetes in pregnancy**	*P = 0.1		*P = 0.05		*P = 1	
No		89(43.8)		52(25.6)		62(30.5)
Yes		0(0.0)		3(75.0)		1(25.0)
**Other medical conditions (hypertension, heart disease, thyroid problem, etc.)**	*P = 0.6		P = 0.3		P = 0.1	
No		81(43.8)		51(27.6)		53(28.6)
Yes		8(36.3)		4(18.2)		10(45.5)

*Fisher’s Exact Test **p value ≤ 0.05**: statistically significant association

^a^ Neurodevelopmental disorders group with n = 89 (43%)

^b^ IDs with comorbidities group with n = 55 (26.6%)

^c^ Other mental/behavioral disorders group n = 63 (30.4%)

## Discussion

It is an undeniable fact that children’s mental health is the key predictor of adults’ mental health. Despite the existence of several studies that address the rates and characteristics of children with psychiatric disorders worldwide, this study is the first investigation of this kind in the Kurdistan Region of Iraq, to the best of our knowledge. According to Alhasnawi et al. and Al-Jawadi et al. [6,7], little is known about the mental health of the Iraqi population, including children, as only a few studies have documented high rates of psychopathology among them. The findings in this research focused on the types and frequencies of the common disorders among the study population of children and adolescents. The study found that neurodevelopmental disorders were the highest in frequency (69.6%). Autism spectrum disorder and intellectual disabilities were the most common of the neurodevelopmental disorders. Other mental/behavioral disorders, including conduct disorders, GAD, MDD, trauma, and stressor-related disorders, conversion disorders, relational problems, psychotic disorders, phobias, obsessions and others, were ranked next with 30.4%. These results were almost compatible with a study performed by Aras et al. in 2014 in Turkey[8].

### Sociodemographic (individual) characteristics

As far as sex is concerned, there were approximately twice as many male patients as female patients, which was predictable, mostly because neurodevelopmental disorders such as autism and intellectual disabilities (which had the highest frequency in our sample) have a sex bias skewed towards boys[9], and many studies performed in similar cultures had the same sex difference[8,10–12].

The present study found that males had higher numbers of ASD, IDs, communication disorders and conduct disorder. A similar result was reported by Newschaffer et al.[13], Sarkhel et al.[14] and others[9,15]. Conversely, females exceeded males in conversion disorder, and a study performed by Ghosh et al.[16] reported a similar outcome.

Regarding age, our results showed that the highest frequency age group was 6–9 years, similar to results reported from Mosul, Iraq in 2007[7]. This is best explained by the fact that at this age, children are introduced to school and face new challenges, making parents more aware of any difference the children might have compared to their peers, and this may make the family more likely to ask for help. This age has been investigated in many studies[17–19] as there are multiple stress factors that were identified in this age range, and these factors can impair children’s and adolescents’ performance and notably increase the risk for dropping out of school[20]

We also found that 6.8% of the patients quit school, mostly due to familial neglect, ignorance, socioeconomic reasons or an inability to keep up with school demands due to their conditions. The unavailability of special education programs for children with neurodevelopmental disorders in the region is the key reason why most of these children cannot continue school, knowing that the regular education school system fails to embrace those with special needs. This percentage of students who drop out of school is considered high compared to other countries such as India, where a study found that school dropouts do not exceed 0.6% in the primary stage[21]. Although the difference of the setting is to be considered, attention must be paid to these numbers, and those who are concerned may need to work more on this issue. Many previous studies have examined seasonal variation in the birth of children with neurodevelopmental and emotional disorders[22,23] with inconsistent results. In our study, the most common season of birth was winter, with more than 34% births, which is consistent with a study performed with autistic children in the Kurdistan region[24].

Most of the patients were Muslims and Kurds, only 17% were Arabs, and most of those were immigrants from nearby areas. Urban residence was by far the most common among the patients (73.4%), and this is understandable, as even in a developed country such as England, research has shown that children in rural areas had fewer problems than those in large urban areas explained by the quality of their schools and lifestyle[25].

The birth order in the family was dominated by the patient being the first child in more than 36% of the cases, followed by the second child with 21.3%. These results were consistent with a study performed in Baghdad[26] and another in New Zealand[27], yet in Pakistan, they had contradictory results, as they found that the 4th child was more prone to psychiatric morbidity due to social and emotional loading and stressors, which affected lower birth orders more[28].

### Sociodemographic (family) characteristics

The mean family size was 6.5±2.8 with a minimum of three members and a maximum of 28 members, and the mode was five members. Most of those were nuclear families with only 13.5% joint families. The residential area ranged from (50–500) m^2^ with a mean of 150±67 m^2^. More than half of them lived in low socioeconomic status. Fathers were mostly school educated, working either as wage earners or employees, with a small percentage (8.7%) of those who did not support their families for various reasons, e.g., being unemployed, divorced or deceased. Similar findings were reported by Al-Jawadi et al. in 2007 in a parallel setting[7].

Mothers were mostly illiterate housewives. Illiteracy was reported in 42% of the mothers. This percentage is higher than what was found in other Iraqi regions such as Mosul and Baghdad [7,26], mostly due to cultural traditions concerning the role of the female and the tradition of early marriage, which is considered a serious and growing issue in the Kurdistan Region of Iraq[29]. These results are significant and need further assessment in the future because mothers’ education may affect the family, pregnancy planning, antenatal care, early diagnosis and plans for treatment[30].

We need additional future studies to elucidate the reasons behind this high percentage of illiteracy and its effects on childhood mental disorders and their management.

The prevalence of consanguinity and rates of first cousin marriage can vary widely within and between populations and communities, depending on ethnicity, religion, culture, and geography. Consanguineous marriages are also practiced among emigrant communities now reside in Europe, North America, and Australia[31]. Marriage between close biological relatives is subjected to substantial criticism and distaste from Western society, reflecting historical and religious prejudice[32] and the fact that it is associated with increased morbidity and mortality. Our data showed that consanguinity was found in 41% of the parents. This percentage was higher than what a study conducted in Qatar in 2016 reported[33], where the consanguinity percentage in the recent generation was 31.5%. This is probably because consanguineous marriage is a common and preferable traditional custom of marriage in the area[34], making the estimated prevalence of such marriages reach 50% and above in Muslim countries of the Middle East[35].

### Pregnancy and birth characteristics

Most of the mothers (74%) had regular antenatal care that variably included monthly visits, vitamin supplements, and immunization when required. This result was higher than what a UNICEF (United Nations Children’s Fund) survey reported from Iraq in 2016 [36] probably owing to the fact of raising awareness and easy accessibility of primary health care centers.

According to Betrán et al.[37], the rate of (C/S) ranges from 6% to 27.2% in the least and the most developed regions, respectively. Latin America and the Caribbean region have the highest C/S rates 40.5%, followed by Northern America 32.3%, Europe 25%, and Asia 19.2%. This study found that rates were higher 26.6%. The reasons for this increase are most likely multifactorial and are affected by many elements, such as the expansion of the private health sector[38].

Breastfeeding is widely recognized as a critical element for public health, not only a matter of lifestyle choice[39,40]. The rates of early initiation of breastfeeding, exclusive breastfeeding and continued breastfeeding to 1 year all varied considerably within the WHO European Region. They found that exclusive breastfeeding rates declined considerably after 4 months and were low in infants under and at 6 months of age[41]. In the present study, the breastfeeding rate in the first 6 months after birth was found to be more than 60%, this number is higher than what was reported by Habib et al.[42], who found that the breastfeeding rate in the first months after birth was 50% in Baghdad in 2007. The increasing numbers are best explained by the economic downturn in the region, as average families cannot afford bottle feeding, making mothers return to the healthy habit of breastfeeding.

### Medical and psychiatric history of the patients

Children with speech and language impairment are an underrepresentation of the broader occurrence of communication disorders[43], especially considering the cooccurrence of communication disorders with other disabilities, resulting in 8% to 12% of preschool populations exhibiting language impairments[44]. Thus, it was not surprising that more than 20% of the complaints of the families who consulted the clinic were speech problems or delayed speech, and many of them were worried about autism. They knew that speech problems are the cardinal sign of this disorder.

On the other hand, we noticed that patients who disclosed their suicidal ideations were less than 1%. This percentage is very low compared to what other studies have reported from Palestine, Lebanon and Morocco[45–47] and considering that suicide is the third leading cause of death among adolescents generally according to Anderson et al.[48]. This low number is probably due to inadequate information and awareness of the families about suicide among children and adolescents, and the fact that adolescent suicidal behavior is a neglected public health issue, especially in developing countries. We wish for more concern and research in the future.

In the pathway of referral, the results showed that most of the patients 39.6% were referred from other clinics, especially the neurology department. A study by Barnett et al.[49] reported that the general referral rate from a physician to another in the United States of America in 2009 did not exceed 9.3%. Our high result may be due to inadequate information and education of people about consulting specific medical specialties in a way that fits their complaints; this makes us think about the need for more awareness and psychoeducation of the society about the features of psychiatric disorders.

Alternative and complementary systems of medicine encompass a broad range of practices that are commonly embedded within contextual cultural milieu, reflecting community beliefs, experiences, religion and spirituality, and they are commonly used by a large number of persons with mental illness[50]. There is well-documented evidence for the increasing widespread use of such systems in the treatment of physical and psychiatric symptoms and disorders within Western populations[51], and in our study population, 42.5% of the patients’ families visited a faith healer before consulting a psychiatrist regardless of their educational and cultural background. Some of those continued to visit the faith healers routinely. A study performed in the region by Rahim et al.[52] reported approximate results of 49%. While in a study conducted in India, they found that 8% of psychiatric patients consult a traditional faith healer at the beginning of their complaints[53]. These comparable high results in the region are probably due to the religious merits of the people who seek solace in Islamic rituals. Thus, we decided to explore this inherited cultural habit and draw more attention to it as it prolongs the duration of the illness and causes more deterioration in mental health during that time before consulting a specialist.

According to the GDS, which we used to estimate the general development of the patients, we found that 29.5% of the scores were within the range for their age expectations, 18.8% scored within 2 SD below their chronological age expectations, placing them in the borderline development group according to the scale, and 51.7% scored more than 2 SD below age, making them delayed compared to their peers.

### Treatment management

Regarding professional management, the study results showed that psychological treatment compared to pharmacological treatment was more trusted and practiced in the child and adolescent psychiatric unit. This trend seems to be consistent with many studies[54–57]. Furthermore, psychological management is more tolerated by patients[58] and their caregivers, especially at early ages.

Regarding the antipsychotic option in the pharmacological line of treatment, atypical antipsychotics were mostly prescribed. The trend of the use of these medications ‘though not fully justified’ seems to be global due to side effects profile and updated guideline instructions [59–61]. For antidepressants, the numbers for selective serotonin reuptake inhibitors (SSRI) and tricyclic antidepressants (TCAs) were relatively close. This result differed from what other studies reported from Germany and Korea[62–63], which revealed that SSRIs are prescribed more in children and adolescents, probably due to the specific guidelines that are followed in each region. Moreover, this dissimilarity might be due to a lack of SSRIs in the outpatient clinic, especially after the economic downturn in the region, which led to the poor availability of all types of medication in the hospital.

We found that stimulants such as methylphenidate were not prescribed, probably due to their unavailability in the hospital and their unaffordable prices outside the hospital. Instead, prescriptions shifted towards atomoxetine, which was available in the hospital during the time of the study.

We found that psychiatrists tended to prescribe a single medication to children, and more than one medication was prescribed in only 17% of the cases, and the remaining 5% received two or more medications. These percentages were convenient when compared to results reported by Olashore et al.[64].

### Association testing

There was no statistically significant association between sex and the three categories of disorders, which contradicts many studies such as Rosenfeld et al.[9], mostly due to small sample size and the unrestricted number of disorders that were involved in the study.

We found that the age of the patients was associated with the diagnoses of each of the three categories of disorders with a statistically significant p value. Similar results have been reported by many studies[65,66]. The educational level of the patients was associated with the three categories of disorders with a statistically significant p value, as in Breslau et al.[67].

There are several mechanisms by which season of birth may affect the developing brain, either directly on the fetus and newborn or indirectly through the mother during pregnancy or the postpartum period. These may include viral or other infections, nutritional factors and vitamin deficiencies according to Shalev et al.[68], who reported an increased risk of ASD among individuals born in August, a comparable result was reported by Livingston et al.[69]. Our findings confirmed the significant association of the season of birth with neurodevelopmental disorders but showed that the risk is more for those born in autumn and to a lesser extent in summer.

Residence was significantly associated with IDs with or without comorbidities and other mental/behavioral disorders, which was consistent with previous studies[25,70].

Socioeconomic status was statistically associated with neurodevelopmental disorders with and without comorbidities. These results are consistent with previous studies[70,71].

Regarding parental age, Croen et al. found that advanced maternal and paternal ages are independently associated with ASD risk[72]. In this study, both paternal and maternal age results showed a nonsignificant association with the 3 categories of the disorders. These results were comparable to a study performed by Larsson HJ et al.[73].

On the other hand, fathers’ and mothers’ education were associated with IDs with comorbidities, which is consistent with a study conducted in Spain[74].

The statistical analysis resulted in a significant association between consanguinity and IDs, which was expected because consanguinity has been considered a risk factor for mental illnesses in many studies[33,35].

The positive family history of mental disorders ratio was 1:1.4, but it was statistically nonsignificant, which contradicts many studies, such as Fullerton et al.[75], probably due to the small sample size and the absence of a focused diagnosis in this study.

Many studies have reported that children born by C/S are approximately 20% more likely to be diagnosed with ASD[76].

Thus, according to our results, delivery by C/S percentage was higher in patients in the neurodevelopmental disorders group in general with a statistically significant association. Yip et al. reported the same result[77].

Normal vaginal delivery (NVD) percentage was higher in patients with IDs and comorbidities with a statistically significant association. This result contradicts other findings[77], probably because more than 20% of the normal vaginal deliveries happened outside a health care facility mostly by uncertified midwives; this makes us suspect birth injuries and related inconveniences in neonatal resuscitation and care. Gestational age was significantly associated with neurodevelopmental disorders, as had been proven by other studies[78,79]. Second-hand smoking during pregnancy was predicted to be related to mood disorders and emotional problems based on Moylan et al.[80]. The same result was found in this study, as smoking in pregnancy was found to be associated significantly with other mental/behavioral disorders.

Diabetes in pregnancy was found to be associated with children’s mental health problems by Li et al.[81]. Our results showed that it was mainly associated with IDs with comorbidities, in a statistically significant manner.

## Limitations

We would like to acknowledge the limitations of our study that included the small sample size due to time constraints, which can explain many statistical inconveniences that might be avoided with larger sample sizes in future studies. Teacher involvement was considered through general and formal notes via school administration, and we hoped for more personal and interventional roles of the teaching and nursery staff.

## Conclusion

This study revealed that the child and adolescent psychiatric disorders in Erbil city are versatile and dominated by neurodevelopmental disorders, and many factors are involved. Statistical results showed that age group, educational status, residence and socioeconomic status were significantly associated with these psychiatric disorders. Father’s and mother’s education and the consanguinity between them were associated with intellectual disabilities. Mode of delivery was associated with neurodevelopmental disorders. Generally, consanguinity was found in 41% of the parents, and delivery by C/S was reported in 26.6% of them. Regardless of their cultural and educational background, 42.5% of the families visited faith healers before consulting a psychiatrist. The study confirmed that the number of patients is much higher than what was expected when this young clinic launched in 2009, and even though it is involved in its patients’ lives in many ways, we still need to fight ancient myths and policies. We need to increase families’ awareness about the signs and symptoms of the common disorders during this critical period of life and about faith healers who proved to be more trusted than health professionals as families considered the doctor as a last resort to solve problems when sometimes it was too late. A new reign of evidence-based methods holds such a big responsibility to those involved, but the prize is worthy: a healthy child and a happy adolescent embraced by a productive community.

## Supporting information

S1 Table Research SPSS.sav. (SAV)

S1 File Research Questionnaire.docx. (DOCX)
