# Supplementary material for: Characteristics of patients attending the child and adolescent psychiatric outpatient clinic in Erbil city
Source: PLoS One. 2019 Feb 28;14(2):e0209418. doi: 10.1371/journal.pone.0209418 (PMC6395034; doi:10.1371/journal.pone.0209418)
Supplement: S1 File — (DOCX) [file pone.0209418.s002.docx]

***Characteristics of Patients Attending the Child and Adolescent***

***Psychiatric Outpatient Clinic in Erbil City***

***(Research Questionnaire )***

**Rank :**

**Name (optional):**

**SOCIODEMOGRAPHIC CHARECTERSTICS**

**Date of birth:**

**Gender: 1.** Male **2.** Female

**Educational status :**  **1**. Preschool age  **2**.Primary school **3**.Intermediate school

**4**.Secondary school **5**.Quit school **6**.Never registered in a school

**Religion :** **1**.Muslim **2**.Christian **3**.Yazidi **4**.Subba

**Ethnicity:**  **1**.Kurdish  **2**.Turkman **3**.Arab **4**. Others

**Residence : 1**. Urban  **2**. Suburban  **3.**Rural **4.**camp

**Family size :**

**His/her order in the family:**

**Residential area : m^2^**

**Socioeconomic Status: 1.** Low  **2.** Medium  **3.**high

**Father’s age: education : occupation :**

**Mother’s age: education: occupation :**

**Consanguinity between the parents : 1.**Yes  **2.**No

**PREGNANCY AND BIRTH**

**Birth weight :** **1**.Below **2,5** kg **2**.Average **(2.5-5)** kg **3**.Above average

**GA at delivery :**

**Mode of delivery:** **1**. NVD  **2**.C/S: causes :

**Stay in premature unit ? 1**. No **2.** Yes .why?

**Start breast feeding ? 1.** No **2.**yes , duration ;

**Developmental History (GDS Scale) : 1.** Normal to his/her age **2.**Delayed to his/her age

**How old was the mother when she gave birth ?** ......years

**History of abortions ? causes?**

**Did the mother have proper and regular Antenatal care (immunization) ? 1.**Yes  **2.**No

**Did she have (during the pregnancy)?**

**1.**Asthma **2.**Allergies **3.** Bleeding **4.**Psychlogical stressors **5.**Smoking (passive ,active)

**6.**Autoimmune disease **7.**Endocrine disease **8.**Infertility requiring medical interventions

**9.**Immunization **10.** Blood transfusion **11.** Anxiety &Depression requiring Tx

**12.**Pregnancy on contraception **13.**X –rays **14.** Other illnesses; specify

**PAST HISTORY**

**Past psychiatric history regardless of the recent one( psychiatric comorbid conditions) :**

**1.** Has no history  **2.** Has a history; specify

**Medical history : 1.** No medical issues **2.** Seizures

**3.** Has other medical issues ,specify

**Family history of mental disorders 1.**No **2**.in Father **3**.in Mother **4**.Relatives; specify

**Number of the previous visits to the outpatient:**

**Visits to the faith healer : 1.**No visits **2.** Yes visited ..mention times

**ABOUT THE RECENT VISIT**

**Source of the referral : 1.**Direct **2.**psych. Clinic **3.**other clinics

**4.** ER **5.** School administration **6.** Dispensary **7.** camps

**Chief complaint that led him/her or the family to the hospital:**

**Duration of this recent illness : Physical complains:**

**Professional primary Diagnosis (DSM5) :** **1**. Intellectual Disability **2**. Communication Disorder **3**.ASD

**4**.ADHD **5**.Specific Learning Disorders **6**.Motor Disorders

**7**. Feeding and Eating Disorders **8**. Elimination Disorders

**9**. Trauma & Stressor Related Disorders **10**. Mood Disorders and Suicide

**11**. Anxiety Disorders **12**.OCD **13**. Early Onset Schizophrenia

**14**. Substance Abuse **15**. Others (identity problems etc..)

**The management done for him: 1.**Investigations **2**. Pharmacological

**3.** Non-pharmacological (counseling or psychotherapy)

**If he/she had pharmacological treatment was it**

Antipsychotic :specify Antidepressants : specify

Anticonvulsants :specify Anxiolytic : specify

Stimulant :specify Others:

**GENERAL DEVELOPMENT SCALE - 70 items**

***Social Scale - 10 items***

Age 1-2

1. Greets people with “Hi” or similar expression. 15m

4. Sometimes says “No” when interfered with. 18m

3. Shows sympathy to other children, tries to help and comfort them 21m

Age 2-3

6. Asks for help in doing things. 2-0

5. Helps a little with household tasks, 2-0

7. Says “I can’t,” “I don’t know,” or “You do it.” 2-6

8. Pays attention well — listens to others. 2-6

9. Apologizes — says “I’m sorry” when he(she) does something wrong. 2-6

Age 3-4

2. Tattles or tells on other children. 3-0

10. Gives directions to other children. 3-0

***Self Help Scale - 10 items***

Age 1-2

50. Lifts a cup to his(her) mouth and drinks. 12m

41. Feeds self with a spoon. 15m

47. Eats with a fork. 18m

42. Eats with a spoon with little spilling. 21m

Age 2-3

49. Opens door by turning knob and pulling. 2-0

43. Washes and dries hands. 2-6

Age 3-4

44. Toilet-trained for urine control and bowel movements. 3-0, 2-6

48. Dresses and undresses without help, except for tying shoelaces. 3-9, 3-6

Age 4-5

45. Buttons one or more buttons. 4-3

46. Buttons a shirt, blouse, or coat, having all the buttons in the correct holes. 5-0, 4-3

***Gross Motor Scale - 10 items***

Age 1-2

81. Walks without help. 12m

83. Throws a ball while standing. 15m

84. Runs. 15m

89. Kicks a ball. 18m

87. Walks up and down stairs alone. 21m

Age 2-3

85. Runs smoothly, turning corners and making sudden stops. 2-0

82. Jumps from steps with feet together. Or used to. 2-3

88. Walks up and down stairs alone, one foot to a step, alternating feet. 2-9

Age 3-4

86. Rides around on tricycle using pedals. 3-0

90. Hops on one foot, at least two times, without support. 3-6

***Fine Motor Scale - 10 items***

Age 1-2

121. Scribbles with crayon or pencil. Or used to. 15m

128. Builds a tower of five or more blocks. 21m

Age 2-3

129. Turns pages of children’s book one page at a time. 2-3, 21m

125. Attempts to cut with small scissors. Or cuts. 2-6

Age 3-4

124. Draws or copies a complete circle 3-3

122. Cuts across paper with scissors from one side to the other. 3-6

Age 4-5

123. Draws recognizable pictures. 4-3

126. Draws or copies a square that has four good corners. ( ). 4-3

127. Cuts with scissors, following a simple outline or pattern. 4-3

130. Draws pictures of people that have at least three parts, such as head, eyes, nose, mouth, hair, body, arms, or legs. 4-6

***Expressive Language Scale - 10 items***

Age 1-2

164. Uses at least five words as names of familiar objects 18m

170. Refers to his(her) things as “my” or “mine.” 21m

Age 2-3

165. Uses at least one of the following words —“me,” “I,” “he,” “she,” “you,” “it.” 2-0

167. Has a vocabulary of 20 or more words. 2-0

161. Talks in sentences at least four words long. 2-6, 2-3

163. Speaks clearly; is understandable most of the time. 2-6

Age 3-4

162. Gives reasons for things, using the word “because....” 3-0

166. Asks questions beginning with “why,” “when,” or “how.” 3-0

169. Talks about things that have happened in detail, describing a series of events, “We went to..., and we.” 3-3

168. Talks in long, complex sentences, ten words or longer. 3-6

***Language Comprehension Scale - 10 items***

Age 1-2

209. Follows simple instructions. 18m

207. Follows two-part instructions, for example,“Go to your room and bring me....” 21m

Age 2-3

206. Responds to simple questions appropriately with “yes” or “no.” 2-0

202. Uses the words “big” and “little.” 2-6

203. Answers questions like “What do you do with a...cracker?...a hat?...a glass?” 2-9

Age 3-4

205. Answers “If..., then?” questions such as “If you get hurt, then what do you do?” 3-0

201. Identifies at least four colors by name correctly. 3-3

204. Answers the questions “What do you do with your...eyes?...ears?” 3-6

Age 4-5

210. Uses —-est words like biggest, strongest, greatest. 4-3

208. When asked, “What is a...?” talks about the group it belongs to, for example, “A horse?”

“Is an animal.” “An orange?” “Is a fruit.” 4-9

***Letters Scale - 5 items***

Age 4-5

251. Prints a few letters or numbers. 4-3

254. Prints first name (or at least four letters). 4-6

Age 5-6

252. Prints two or more simple words from a copy. 5-3

Age 6

255. Recognizes and names all the letters in the alphabet. 6-0

253. Reads four or more words. 6+

***Numbers Scale- 5 items***

Age 3-4

257. Talks about things, comparing one to another, for example, says “This one is bigger, ...heavier, etc.” 3-0

259. Tells when one object is longer or shorter than another object. 3-6

Age 4-5

256. Counts ten or more objects 4-0

Age 5-6

258. Recites numbers in order from 1 to 30. 5-9

Age 6

260. Answers arithmetic questions such as “How much is 2+2? 1+4? 3+6?” 6+

**Date : / / 2017**

**Mobile no.**
